# Cortical Inhibitory Imbalance in Functional Paralysis

**DOI:** 10.3389/fnhum.2020.00153

**Published:** 2020-05-07

**Authors:** Alberto Benussi, Enrico Premi, Valentina Cantoni, Silvia Compostella, Eugenio Magni, Nicola Gilberti, Veronica Vergani, Ilenia Delrio, Massimo Gamba, Raffaella Spezi, Angelo Costa, Michele Tinazzi, Alessandro Padovani, Barbara Borroni, Mauro Magoni

**Affiliations:** ^1^Neurology Unit, Department of Clinical and Experimental Sciences, University of Brescia, Brescia, Italy; ^2^Stroke Unit, Azienda Socio Sanitaria Territoriale Spedali Civili, Brescia, Italy; ^3^U.O. Neurologia, Fondazione Poliambulanza Hospital, Brescia, Italy; ^4^Neurology Unit, Movement Disorders Division, Department of Neurosciences, Biomedicine and Movement Sciences, University of Verona, Verona, Italy

**Keywords:** functional neurological disorders, functional paralysis, transcranial magnetic stimulation, short interval intracortical inhibition, motor threshold

## Abstract

**Background:**

Functional neurological disorders are characterized by neurological symptoms that have no identifiable pathology and little is known about their underlying pathophysiology.

**Objectives:**

To analyze motor cortex excitability and intracortical inhibitory and excitatory circuits’ imbalance in patients with flaccid functional weakness.

**Methods:**

Twenty-one consecutive patients with acute onset of flaccid functional weakness were recruited. Single and paired-pulse transcranial magnetic stimulation (TMS) protocols were used to analyze resting motor thresholds (RMT) and intracortical inhibitory (short interval intracortical inhibition – SICI) and excitatory (intracortical facilitation – ICF) circuits’ imbalance between the affected and non-affected motor cortices.

**Results:**

We observed a significant increase in RMT and SICI in the affected motor cortex (*p* < 0.001), but not for ICF, compared to the contralateral unaffected side.

**Conclusion:**

This study extends current knowledge of functional weakness, arguing for a specific central nervous system abnormality which may be involved in the symptoms’ pathophysiology.

## Introduction

Functional neurological disorders (FND) are characterized by neurological symptoms that have no identifiable, responsible pathology (American Psychiatric Association, 2013; Edwards et al., 2014; Roelofs et al., 2019; Baizabal-Carvallo and Jankovic, 2020). Functional disorders are common in neurological practice, accounting up to 15% of new referrals in neurology clinics (Stone et al., 2010). Despite their common occurrence, little is known about the underlying pathophysiology and treatment, also considering that they have a poor prognosis at long term follow-up, with high levels of physical disability (Espay et al., 2006; Avanzino et al., 2008; Quartarone et al., 2009). Individuals with FND have traditionally been left in a therapeutic void, with neither neurologist nor psychiatrics able to provide adequate strategies for improvement (Pringsheim and Edwards, 2017).

Recent studies have shown that patients with FNDs are not different psychologically from individuals with neurologic movement disorders, and that a relevant proportion of individuals with FND have no psychopathology detectable on symptom screening measures (van der Hoeven et al., 2015). These symptoms are thought to result from a distorted mind-body relationship in self-awareness, with a functional mismatch between beliefs/expectations and sensory information related to the symptom (Edwards et al., 2013), rather than an intention to obtain earning or help (Espay et al., 2018). However, researchers are continuing to explore the psychological underpinnings of FND, still debating whether to support the Freudian hypothesis of conversion or to embrace a neurologic (organic) explanation for the disorder (Pringsheim and Edwards, 2017).

Among FNDs, a great proportion is determined by patients presenting with sudden weakness, mimicking an acute stroke (stroke-mimic) (Carson et al., 2000). In functional weakness and paralysis, the motor system, from motor cortex to muscle, seems to be unaffected. To date, the diagnosis is based exclusively on ruling out organic disorders, while we still lack an objective measure of disease.

A possible marker could reside in the functional evaluation of the motor cortex, but mixed results have been reported regarding motor cortex excitability and intracortical inhibitory circuits, evaluated with transcranial magnetic stimulation (TMS) (Premi et al., 2017). These studies were performed in small groups of patients, including both flaccid and spastic-dystonic paralysis.

TMS has already been proven useful to assess non-invasively and *in vivo* several inhibitory and excitatory intracortical circuits, as well as several parameters of cortical plasticity (Rossini et al., 2015). These techniques have been successfully applied in several studies, which have included stroke, movement disorders and neurodegenerative diseases (Motta et al., 2018; Koch et al., 2019; Benussi et al., 2020).

Abnormalities in frontal, parietal and limbic influences on the motor system have recently emerged (Kanaan et al., 2007; Stone et al., 2007; Cojan et al., 2009; Demartini et al., 2019), suggesting for a top-down inhibition of the motor system causing weakness or paralysis (Hallett, 2016). In particular, these studies have provided novel insights into the possible neural mechanisms involved in functional paralysis, by showing that unilateral paralysis was associated not only with a suppressed activation of the motor cortex during attempted movements but also with changes in its functional connectivity, including greater recruitment of the precuneus and ventromedial prefrontal cortex regions that are critical for accessing self-related representations and memories. Moreover, no evidence has emerged that brain regions normally implicated in conscious motor inhibition (such as the inferior frontal gyrus) are responsible for the paralysis (Cojan et al., 2009).

In this view, FNDs would represent a proper neurological disorder, beyond the possible presence of a specific psychological or psychiatric trait (Edwards et al., 2013).

The objective of this study was to evaluate possible abnormalities in motor cortex excitability between the affected and unaffected motor cortices in a relatively large cohort of well characterized patients with flaccid functional weakness, mimicking an acute stroke, by applying paired-pulse TMS protocols measuring short interval intracortical inhibition (SICI) and intracortical facilitation (ICF).

## Methods

### Participants

Twenty-one patients were recruited from the Stroke Unit, ASST Spedali Civili Hospital, Brescia, and from the Neurology Unit, Fondazione Poliambulanza Hospital, Brescia, Italy with an acute onset functional paralysis (flaccid hemiparesis, 7 right-sided, 14 left-sided), admitted for a clinical suspect of ischemic stroke with acute hemiparesis.

Brain CT scan was unremarkable and some patients (*n* = 11) were treated with recombinant tissue plasminogen activator if clinical and temporal inclusion criteria were satisfied (Powers et al., 2019).

At 24 h follow-up, brain 1.5T or 3T MRI scans with T1, T2, T2-Fluid Attenuated Inversion Recovery (FLARI) and Diffusion Weighted Images (DWI) sequences resulted unremarkable and ruled out a cerebrovascular event or any other abnormality that could have possible explained the symptomatology.

At discharge (range: 4–7 days after admission), global neurological examinations showed a persistent hemiparesis. At follow-up (mean disease duration: 22.5 ± 31.5 months), considering the persistency of clinical symptoms, brain and cervical spine MRI, and EMG-ENG were performed in all patients to exclude lesions in the cervical spinal cord (i.e., myelopathy) or peripheral nerves. All tests were unremarkable and excluded an organic lesion. Central motor conduction time (CMCT) was not evaluated in all patients, thus possible subclinical myelopathies could have been undiagnosed.

The functional etiology was also defined according to the Carson scale (Carson et al., 2000) (1 = not at all; 2 = somewhat; 3 = largely; 4 = completely) based on the assessment of the premorbid psychological status by Minnesota Multiphasic Personality Inventory 2 (MMPI-2) (Tellegen and Ben-Porath, 1993) and the Italian version of the Symptom Rating Test (Fava et al., 1983). Functional etiology was confirmed by unremarkable instrumental examination, including the assessment of the premorbid psychological status, which supported the presence of a largely (*n* = 13) or completely (*n* = 8) functional etiology, in line with the Carson scale (Carson et al., 2000). None of the patients were excluded and all met criteria for a functional etiology.

Patients were screened for depression using the Zung Self-Rating Depression Scale (Zung, 1965).

None of the patients were being treated with drugs that could have altered the cerebral cortex excitability at TMS evaluation.

Informed consent was acquired from all participants in accordance to the Declaration of Helsinki. The local ethics committee of the Brescia Hospital approved the present study (05.19.2015, #NP1965).

70 healthy subjects (age 44.9 ± 16.5) who underwent TMS on the left motor cortex were included as a control group.

### Transcranial Magnetic Stimulation Variables and Protocols

A TMS figure-of-eight coil (each loop diameter 70 mM – D70^2^ coil) connected to a monophasic Magstim Bistim^2^ system (Magstim Company, Oxford, United Kingdom) was employed for all TMS paradigms, as previously reported (Benussi et al., 2018a). Motor evoked potentials (MEPs) were recorded from the right and left first dorsal interosseous (FDI) muscles through surface Ag/AgCl electrodes placed in a belly-tendon montage and acquired using a Biopac MP-150 electromyograph (BIOPAC Systems Inc., Santa Barbara, CA, United States). Responses were amplified and filtered at 20 Hz and 2 kHz with a sampling rate of 5 kHz and recorded on a personal computer for offline elaboration (AcqKnowledge 4.1, BIOPAC Systems Inc., Santa Barbara, CA, United States).

Resting motor threshold (RMT) was determined on both motor cortices as the minimum intensity of the stimulator required to elicit motor evoked potentials (MEPs) with a 50 μV amplitude in 50% of 10 consecutive trails, recorded form the right or left first dorsal interosseous muscles during full relaxation (Rossini et al., 2015). Moreover, MEP latencies were measured on both sides at an intensity of 120% RMT.

SICI and ICF were studied using a paired-pulse technique, employing a conditioning-test design. For all paradigms, the test stimulus (TS) was adjusted to evoke a MEP of ∼1 mv amplitude in the right and left FDI muscles (Benussi et al., 2018b).

The conditioning stimulus (CS) was adjusted at 70% of the RMT, employing multiple interstimulus intervals (ISIs), including 1, 2, 3 ms for SICI and 7, 10, 15 ms for ICF (Kujirai et al., 1993; Ziemann et al., 1996). For each ISI and for each protocol, ten different paired CS-TS stimuli and fourteen control TS stimuli were delivered in all participants in a pseudo randomized sequence, with an inter trial interval of 5 s (± 10%). Patients were stimulated on the left side first in ∼50% trails in order to reduce possible effects of attention and adaptation to the TMS pulses and thus on motor cortex excitability measures.

The conditioned MEP amplitude, evoked after delivering a paired CS-TS stimulus, was expressed as percentage of the average control MEP amplitude. Audio-visual feedback was provided to ensure muscle relaxation during the entire experiment and trials were rejected if electromyographic activity was greater that 100 μV in the 250 ms before TMS stimulus application. Less that 2% of trials were discarded for each protocol. All of the participants were capable of following instructions and reaching complete muscle relaxation; if, however, the data was corrupted by patient movement, the protocol was restarted and the initial recording was rejected.

### Statistical Analysis

TMS evoked responses were compared using one-way repeated measures ANOVA (for RMT and MEP latency) or two-way repeated measures ANOVA (for SICI and ICF) with SIDE (affected vs. unaffected) and ISI (1, 2, 3, 7, 10, 15 ms) as within-subjects factor. If a significant main effect was observed, group differences were evaluated with *post hoc* tests (*p-*values are reported after Bonferroni correction for multiple comparisons). Mauchly’s test was used to check for sphericity violation. Pearson’s correlation co-efficient was used to investigate any association between differences in RMT and SICI scores between sides and with NIH Stroke Scale scores (Pezzella et al., 2009).

Data analyses were carried out using SPSS 21.0 software.

## Results

### Participants

Demographic and clinical characteristics are reported in Table 1. We did not observe a significant association between handedness and side of the hemiparesis, as assessed by Fisher’s exact test (*p* = 0.533). NIH Stroke Scale scores were 3.8 ± 1.4 and 2.4 ± 0.7 at onset and at TMS, respectively.

**TABLE 1:** Demographic and clinical characteristics of included patients.

	Functional neurological disorders	Healthy controls
Patients (n)	20	70
Age (years)	43.5 ± 11.9	44.9 ± 16.5
Gender (% female)	95.0%^∗^	62.9%
Education (years)	11.8 ± 2.7	12.8 ± 4.3
Duration (months)	22.4 ± 32.5	–
Hemiparesis side (% right)	35.0%	–
Handedness (% right)	95.0%	87.1%
NIH Stroke Scale at onset	3.8 ± 1.4	–
NIH Stroke Scale at TMS	2.4 ± 0.7	–
Zung Self-rating Depression Scale	40.6 ± 13.3	–

Demographic and clinical characteristics are expressed as mean ± SD or %, as appropriate. *Significant p-values compared to healthy controls for one-way ANOVA (post hoc tests with Bonferroni correction for multiple comparisons) or Fisher’s exact test, where appropriate.

### Transcranial Magnetic Stimulation

In one patient, RMT could not be measured on the affected side because the motor cortex was unexcitable at 80% of the maximum stimulator output (% MSO) (intensities between 80 and 100% MSO were not tested for patient discomfort), and was excluded from analysis.

No significant differences in MEP latencies between sides were observed, *F*_(1,__19__)_ = 0.42, *p* = 0.524, partial η^2^ = 0.02 (see Table 2).

**TABLE 2:** Neurophysiological characteristics of included patients.

Neurophysiological measures	Affected side	Unaffected side	Healthy controls
MEP latencies (ms)	23.4 ± 0.9	23.5 ± 0.9	–
RMT (% MSO)	49.1 ± 9.0^∗^	43.3 ± 7.3	45.1 ± 9.3
1 mV (% MSO)	59.3 ± 13.3^∗^	51.7 ± 9.5	53.8 ± 12.0
Average SICI (ratio)	0.18 ± 0.05^∗^	0.39 ± 0.10^#^	0.22 ± 0.11
Average ICF (ratio)	1.40 ± 0.30	1.44 ± 0.28	1.50 ± 0.2

**RMT and 1 mV are expressed as ratio of the MSO; SICI and ICF are represented as ratio of mean motor evoked potential (MEP) amplitude related to the control MEP. RMT, resting motor threshold; MSO, percentage of maximal stimulator output; SICI, mean short interval intracortical inhibition (1, 2, 3 ms) expressed as ratio of unconditioned response; ICF, mean intracortical facilitation (7, 10, 15 ms) expressed as ratio of unconditioned response; MEP, motor evoked potential. ^∗^Significant p-values for one-way ANOVA compared to the unaffected side (post hoc tests with Bonferroni correction for multiple comparisons). ^#^Significant p-values for one-way ANOVA compared to the healthy controls (post hoc tests with Bonferroni correction for multiple comparisons).**

One-way repeated measures ANOVA showed a significant difference in RMT scores between the affected and the unaffected side, *F*_(1,__19__)_ = 26.62, *p* < 0.001, partial η^2^ = 0.58, with a significantly increased RMT in the affected motor cortex (49.1 ± 9.0% MSO) compared to the unaffected one (43.3 ± 7.3% MSO) (see Table 2).

RMT was significantly increased in the affected side compared to healthy controls, *F*_(1,__89)_ = 5.07, *p* = 0.027, partial η^2^ = 0.05, but not in the unaffected side, *F*_(1,__89)_ = 0.20, *p* = 0.656, partial η^2^ < 0.01 (see Table 2).

**FIGURE 1:**
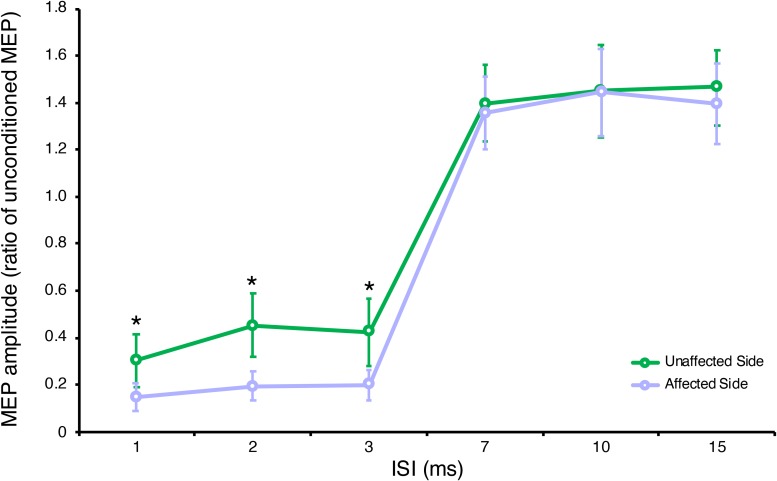
Neurophysiological parameters in the affected and unaffected sides. Short-interval intracortical inhibition (SICI) at ISI 1, 2, 3 ms and intracortical facilitation (ICF) at ISI 7, 10, 15 ms. Data are represented as a ratio to the unconditioned MEP amplitude; error bars represent standard errors. MEP, motor evoked potential; ISI, inter stimulus interval. *p < 0.05 vs. contralateral side using one-way ANOVA (*post hoc* tests with Bonferroni correction for multiple comparisons).

Two-way repeated measures ANOVA highlighted a significant ISI **×** GROUP interaction for SICI-ICF, *F*_(__5,__95__)_ = 3.87, *p* = 0.003, partial η^2^ = 0.17. *Post hoc* tests, with Bonferroni corrections for multiple comparisons, showed a significant difference in MEP amplitudes between the affected and unaffected side at ISI 1, 2, 3 ms (for all *p* < 0.001) but not at ISI 7, 10, 15 ms (for all *p* > 0.005) (see Figure 1), with a significantly increased SICI in the affected (average 0.18 ± 0.05) compared to the unaffected side (average 0.39 ± 0.10) (see Table 2).

There was no significant correlation between the difference in RMT measures between sides and the difference in average SICI between sides (*r* = 0.149, *p* = 0.531).

We observed a significant correlation between NIH Stroke Scale scores and average SICI in the affected side (*r* = 0.601, *p* = 0.005), but not in the unaffected side, nor with RMT or SICI (both affected and unaffected sides), all *p* > 0.05.

Compared to healthy controls, we observed a significant ISI × GROUP interaction for SICI-ICF in the affected, *F*_(6,__528)_ = 8.73, *p* < 0.001, partial η^2^ = 0.09, and unaffected sides, *F*_(6,__528)_ = 3.41, *p* = 0.003, partial η^2^ = 0.04. *Post hoc* tests, with Bonferroni corrections for multiple comparisons, showed a significant increase in SICI at ISI 2 ms (*p* = 0.022) in the affected side compared to healthy controls, and a significant decrease in SICI at ISI 1, 2, 3 ms (all *p* < 0.001) in the unaffected side compared to healthy controls.

## Discussion

In the present study, carried out in a well characterized group of patients with long-lasting flaccid functional paralysis, we observed an imbalance of intracortical inhibition between the affected and unaffected motor cortices. In particular we observed an increase in motor threshold and intracortical inhibition, evaluated with the SICI paired-pulse TMS protocol. SICI is thought to reflect short-lasting post-synaptic inhibition mediated through GABAergic interneurons, modulating the activity of the corticospinal output. In this case, an increase in SICI could reflect an increase of inhibitory intracortical circuits affecting corticospinal excitability, possibly inducing a decrease in voluntary movements.

Previous studies have shown that patients with functional paralysis show a paradoxical suppression of the MEP amplitude after motor imagery in the affected side, opposite to what is observed in healthy subjects, suggesting for a disturbed control of voluntary movements. Conversely, action observation has shown to induce an increase in motor cortex excitability similar to healthy controls (Liepert et al., 2011). One of these studies, performed on 3 patients with flaccid paralysis, did not observe any significant differences in SICI or ICF between the affected and unaffected side (Liepert et al., 2008). Subsequently, another study performed on 4 patients with functional paralysis showed a significant increase in SICI and a non-significant increase in RMT in the affected side compared to healthy controls (Premi et al., 2017).

Several studies using functional neuroimaging have now shown that frontal areas are dysfunctional and particularly strongly connected to the affected motor cortex (Cojan et al., 2009; Nowak and Fink, 2009; Hallett, 2016. Interestingly, long-lasting functional paralysis has been associated with greater cortical thickness/density in motor areas, suggesting that functional brain abnormalities lead to structural changes when disease is sustained over time (Aybek et al., 2014).

Interestingly, patients with depression exhibit a significant interhemispheric difference in motor cortex excitability, an imbalanced inhibitory and excitatory intracortical circuitry, and an impaired long-term potentiation-like response to paired-associative transcranial magnetic stimulation, reinforcing the possible parallelism between FND and psychological comorbidity (Cantone et al., 2017).

In line with previous studies on FND, the majority of patients in this study were represented by females. There is currently no clear scientific explanation for the differences in gender prevalence favoring females but it can be argued that neurobiological, hormonal, cultural, social, and previous history of psychological or sexual trauma may be relevant contributors (Baizabal-Carvallo and Jankovic, 2020; Edwards and Aybek, 2020).

Interestingly, in one patient we could not evoke a reliable MEP on the affected motor cortex, which is somewhat contradictory with previous studies, in which patients with psychogenic paralysis had normal MEPs, confirming the physiological integrity of motor fibers in the corticospinal tract, anterior roots and plexuses (Cantello et al., 2001).

Other than evaluating intracortical circuits and corticospinal tract integrity, TMS may be also used as a therapeutic option in patients with FND. It has been demonstrated that TMS may exert a therapeutic effect via genuine neuromodulation, via non-specific placebo effects and by demonstrating, through its immediate effects on the motor system (i.e., movement in a “paretic” limb), that symptom improvement is possible, thus directly changing higher level beliefs that may be responsible for the maintenance of the disorder (Pollak et al., 2014; Schönfeldt-Lecuona et al., 2016; Garcin et al., 2017).

We acknowledge that this study entails some limitations. Firstly, it is not known if these modifications in cortical excitability are the cause or consequence of the functional disorder. To this, further studies should assess cortical excitability in the acute phase of symptoms’ onset. Supporting these findings and arguing for a causal role of increased SICI in functional hemiparesis are previous findings observed at only 1 month after symptom onset in patients with functional paralysis (Premi et al., 2017). Moreover, these findings should be compared with patients with organic hemiparesis (i.e., after stroke), in order to shed further light on disease pathophysiology.

Moreover, it would have been interesting to assess the excitability of the motor cortex with further measures, primarily the input–output (IO) curves and TMS mapping. The IO curves can be easily measured by plotting the amplitude of motor evoked potentials (MEP) against a range of different stimulus intensities, and are sensitive measures of changes in neuronal system excitability (Ridding and Rothwell, 1997). TMS mapping, i.e., the assessing of the area (number of scalp positions from which a MEP can be elicited) and volume (the sum of the averaged MEP amplitudes for each excitable scalp site) of the cortical representation of a target muscle is relatively more time consuming but provides interesting information; in stroke, for example, hand muscles of the paretic side are underrepresented (Marconi et al., 2007). Another relevant aspect could have been to investigate interhemispheric connectivity between the two motor cortices, using a paired coil method to test interhemispheric inhibition (IHI) (Ferbert et al., 1992). In stroke survivors, a disruption of IHI from the unaffected to the affected motor cortex via glutamatergic transcallosal fibers can interfere with motor performance of the paretic limb (McDonnell and Stinear, 2017). Moreover, EEG analysis should be performed in functional paralysis, possibly providing novel aspects in disease pathophysiology (Cojan et al., 2009). Finally, the small sample size and the gender imbalance may preclude generalization of these results to larger populations.

## Conclusion

The asymmetry/imbalance of SICI between the affected and unaffected motor cortices could represent a potential tool to support the diagnosis of FND in flaccid hemiparesis. To this, further studies on larger samples are warranted, combining clinical, electrophysiological and neuroimaging approaches for a more comprehensive assessment.

## Data Availability Statement

The datasets generated for this study are available on request to the corresponding author.

## Ethics Statement

The studies involving human participants were reviewed and approved by the local ethics committee of the Brescia Hospital. The patients/participants provided their written informed consent to participate in this study.

## Author Contributions

(1) Research project: (A) EP, AB, and BB: conception; (B) EP, AB, BB, and MM: organization; (C) EP, AB, VC, SC, EM, NG, VV, ID, MG, RS, AC, MT, AP, BB, and MM: execution. (2) Statistical analysis: (A) EP and AB: design; (B) EP and AB: execution; (C) EP, AB, and BB: review and critique. (3) Manuscript preparation: (A) EP, AB, and BB: writing of the first draft; (B) EP, AB, VC, SC, EM, NG, VV, ID, MG, RS, AC, MT, AP, BB, and MM: review and critique.

## Conflict of Interest

The authors declare that the research was conducted in the absence of any commercial or financial relationships that could be construed as a potential conflict of interest.
